# Supplementary material for: Cross-National Associations between Age at Marriage and Intimate Partner Violence among Young Women: An Analysis of Demographic and Health Surveys from 48 Countries
Source: Int J Environ Res Public Health. 2023 Feb 12;20(4):3218. doi: 10.3390/ijerph20043218 (PMC9967946; doi:10.3390/ijerph20043218)
Supplement: Supplementary file 1 [file ijerph-20-03218-s001.zip › ijerph-2097927-supplementary.pdf]

Supplementary Materials

Pooled multilevel logistic regression models – women aged 20 to 24 years

We used fractional polynomials with two powers to determine the best parametrization for the age at the first marriage in the pooled multilevel logistic regression models. The two powers with the best fit selected by the fp command in Stata are presented in Supplementary Table 1. The default powers tested by Stata are -2, -1, -0.5, 0, 0.5, 1, 2, and 3. Further information about the fp command is available in https://www.stata.com/manuals13/rfp.pdf. For all models, 55,886 women had information available for all variables. The average number of women in each country was 1,164, ranging from 161 to 8,694. Supplementary Table 2 shows the regression coefficients for each power.

Supplementary Table S1. Selected powers for the pooled multilevel logistic regression models for women aged 20 to 24 years. Each model corresponds to one type of IPV.

IPV type	First power	Second power
Physical	$(\text{age at first marriage})^2$	$(\text{age at first marriage})^3$
Sexual	$(\text{age at first marriage})^{-1}$	$(\text{age at first marriage})^{-1} \times \ln(\text{age at first marriage})$
Psychological	$(\text{age at first marriage})^3$	$(\text{age at first marriage})^3 \times \ln(\text{age at first marriage})$

Supplementary Table S2. Regression coefficients for the selected powers of the pooled multilevel logistic regression models for women aged 20 to 24 years. Each model corresponds to one type of IPV.

IPV type	Coefficient of the first power (95% CI)	Coefficient of the second power (95% CI)
Physical	0.008 (-0.002; 0.019)	-0.000 (-0.001; -0.000)
Sexual	-151.7 (-261.1; -42.4)	96.2 (34.7; 157.8)
Psychological	0.001 (-0.000; 0.001)	-0.000 (-0.000; 0.000)

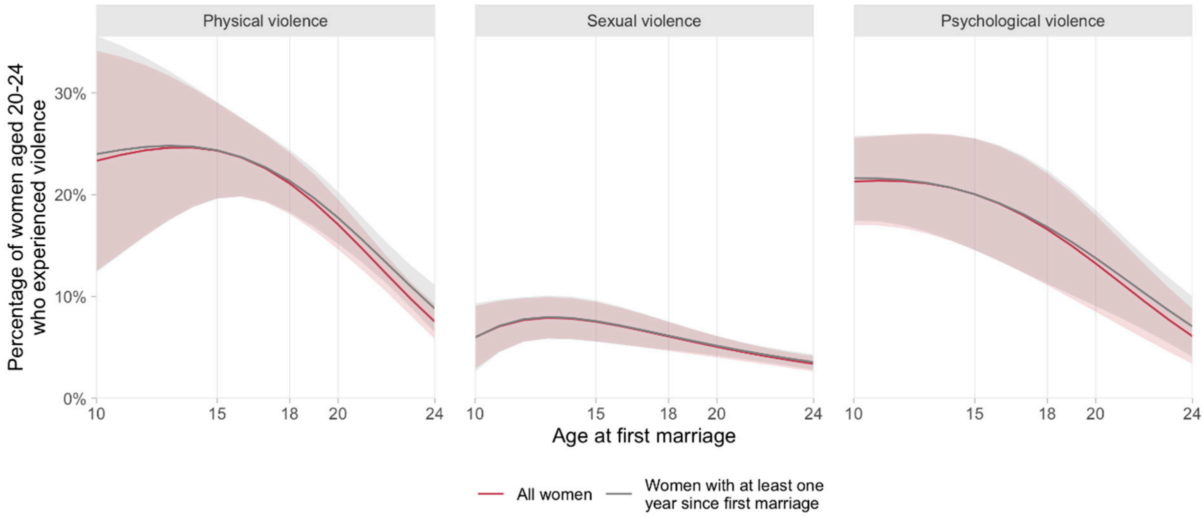

Supplementary Figure S1. Sensitivity analysis comparing the results for all women with women who had been married for at least one year.

Supplementary Table S3. Country specific differences on the prevalence of IPV.

Country	Physical IPV				Sexual IPV				Psychological IPV			
	Difference	95%CI		N women who reported IPV	Difference	95%CI		N women who reported IPV	Difference	95%CI		N women who reported IPV
		Lower limit	Upper limit			Lower limit	Upper limit			Lower limit	Upper limit	
**Afghanistan**	27.9	16.3	39.5	1446	4.7	1.6	7.8	254	23.6	14.4	32.8	1024
**Angola**	9.5	-7.0	25.9	432	3.2	-12.2	18.5	143	8.9	-6.9	24.8	352
**Armenia**	16.1	-25.0	57.3	7	0.0	0.0	0.0	----	15.4	-12.4	43.2	18
**Benin**	9.9	3.7	16.1	60	-0.5	-8.5	7.5	42	13.1	-5.5	31.8	179
**Burkina_Faso**	11.5	7.3	15.7	197	2.3	0.9	3.7	35	7.6	3.8	11.4	154
**Burundi**	12.3	-0.8	25.3	267	3.6	-9.9	17.0	253	6.3	-7.7	20.3	220
**Cambodia**	9.1	2.7	15.5	38	8.3	-0.2	16.7	15	14.2	5.7	22.7	61
**Cameroon**	-2.5	-19.9	15.0	145	0.7	-10.6	11.9	54	9.6	-5.1	24.2	137
**Chad**	-13.1	-34.0	7.8	99	2.3	-13.2	17.9	50	-12.8	-35.9	10.3	106
**Colombia**	17.0	7.2	26.8	1095	6.0	2.9	9.1	149	24.5	15.9	33.2	973
**Comoros**	-5.8	-42.8	31.1	29	-24.8	-74.7	25.0	8	3.6	-3.1	10.2	32
**Congo_Democratic_Republic**	22.9	6.5	39.2	410	3.4	-27.3	34.2	234	23.3	8.2	38.5	362
**Cote_dIvoire**	1.0	-13.4	15.5	212	4.5	-2.5	11.5	49	-7.4	-21.3	6.5	122
**Dominican_Republic**	12.8	3.1	22.5	142	1.9	-3.7	7.4	42	21.8	7.2	36.5	253
**Egypt**	10.1	-1.3	21.6	164	-0.4	-5.9	5.0	31	12.8	4.0	21.5	122
**Ethiopia**	4.2	-15.6	23.9	130	5.4	-6.0	16.9	41	-15.1	-63.7	33.5	145
**Gabon**	14.7	-7.5	36.8	239	7.3	-5.2	19.8	72	30.4	16.6	44.3	180
**Gambia**	6.7	2.4	11.0	60	0.0	-1.1	1.0	11	5.0	-0.7	10.7	71
**Guatemala**	4.2	-6.5	15.0	101	2.9	-2.1	7.9	25	14.2	1.3	27.1	174
**Haiti**	17.2	5.4	29.0	68	-4.9	-18.4	8.6	43	0.6	-20.4	21.6	100
**Honduras**	9.2	2.4	16.1	258	2.1	-1.2	5.3	62	23.9	17.1	30.8	469
**India**	18.0	13.1	22.8	1923	2.2	0.0	4.3	456	10.0	6.8	13.3	846

**Jordan**	3.7	-11.8	19.2	66	0.2	-2.7	3.1	15	16.1	-2.9	35.1	97
**Kenya**	20.1	3.4	36.7	169	6.2	-6.1	18.5	63	30.8	20.7	40.8	167
**Kyrgyzstan**	4.9	-4.5	14.3	98	6.1	-8.2	20.4	19	-1.2	-6.9	4.5	47
**Liberia**	-16.2	-53.1	20.7	168	-14.8	-46.3	16.6	33	-14.9	-54.4	24.6	149
**Malawi**	16.8	7.9	25.7	199	19.9	14.5	25.4	187	10.4	-6.0	26.9	245
**Maldives**	9.2	-40.9	59.2	21	0.0	0.0	0.0	4	-0.3	-2.2	1.6	22
**Mali**	3.8	-15.2	22.9	110	-4.3	-32.1	23.6	49	14.9	-0.5	30.3	157
**Mozambique**	9.4	-3.0	21.7	323	0.9	-6.8	8.7	92	-10.9	-37.2	15.5	384
**Myanmar**	9.5	-3.8	22.8	48	1.8	-4.2	7.7	15	6.5	-0.5	13.5	48
**Namibia**	6.3	-18.8	31.3	42	2.5	-16.7	21.6	8	-6.2	-43.4	30.9	37
**Nepal**	8.0	-10.5	26.5	65	1.8	-13.1	16.8	36	4.7	-3.6	13.0	39
**Nigeria**	13.2	8.7	17.6	204	11.0	6.0	16.0	100	21.3	9.9	32.7	385
**Pakistan**	9.1	-1.2	19.5	86	3.5	-5.1	12.2	17	23.9	13.2	34.6	120
**Papua_New_Guinea**	44.6	29.8	59.4	290	18.4	1.0	35.9	143	46.6	33.3	60.0	270
**Peru**	4.4	-8.9	17.7	395	3.5	0.2	6.9	77	7.0	2.7	11.4	292
**Philippines**	6.0	-1.2	13.3	114	-2.4	-19.5	14.8	46	2.5	-7.0	11.9	129
**Rwanda**	30.5	-2.2	63.1	49	16.8	-12.1	45.7	23	27.6	-9.6	64.8	46
**Senegal**	7.7	2.3	13.0	18	0.3	-9.5	10.2	8	10.0	-2.7	22.8	16
**Sierra_Leone**	5.1	-15.9	26.1	263	-0.4	-10.2	9.4	40	9.8	-18.0	37.7	262
**Tajikistan**	15.1	-0.8	31.1	128	2.2	-3.6	8.1	10	6.0	-7.0	19.0	88
**Tanzania**	30.3	21.4	39.2	380	11.1	3.5	18.6	151	25.5	14.9	36.2	349
**Timor_Leste**	-25.9	-60.7	8.8	137	-7.9	-28.7	12.9	24	6.6	-0.8	13.9	36
**Togo**	6.7	-1.4	14.7	102	-21.3	-52.2	9.6	43	1.8	-12.1	15.6	181
**Uganda**	21.4	12.2	30.6	439	8.9	-4.4	22.2	293	20.8	9.4	32.2	507
**Zambia**	14.7	3.1	26.3	326	11.1	3.3	18.9	164	15.5	4.6	26.4	308
**Zimbabwe**	4.4	-8.9	17.8	216	10.6	1.8	19.3	101	16.1	3.2	29.0	256
**Pooled**	**16.9**	**13.3**	**20.5**	**11978**	**5.9**	**3.4**	**8.4**	**3831**	**10.6**	**7.7**	**13.6**	**10737**

*Prevalence difference derived from logistic regression with fractional polynomials, controlling for covariates. We calculated the average adjusted IPV prevalence for girls first married at 15 years of age and 20 years of age with margins command.

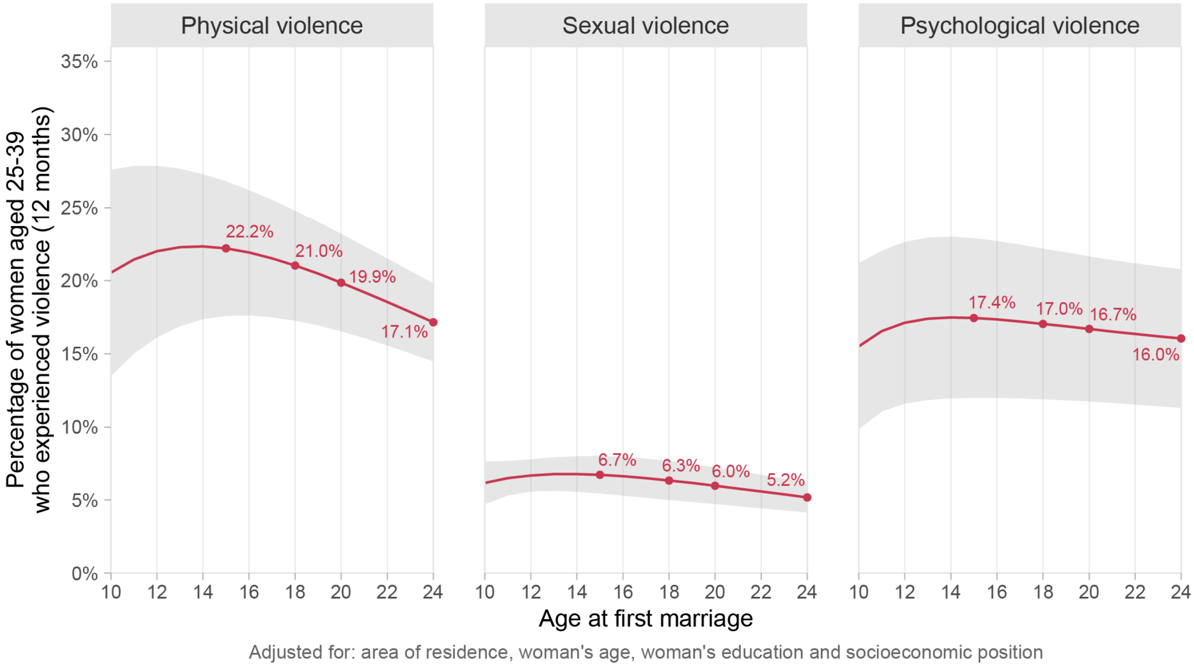

Supplementary Figure S2. Average adjusted IPV prevalence according to age at first marriage for women aged 25 to 39 years. Pooled results for 48 low- and middle-income countries obtained from a multilevel logistic regression model. The red line represents the continuous IPV estimate from ages 10 to 24 years at first marriage. The grey area represents the respective confidence interval. IPV estimates for the ages 15, 18, 20, and 24 years are highlighted.

Pooled multilevel logistic regression models – women aged 25 to 39 years

We repeated the same analysis for women aged 25 to 39 years. The two powers with the best fit selected by the fp command in Stata are presented in Supplementary Table 3. For all models, 205,927 women had information available for all variables. The average number of women in each country was 4,209, ranging from 770 to 37,449. Supplementary Table 4 shows the regression coefficients for each power. Supplementary Figure 2 shows the average adjusted IPV prevalence according to age at first marriage for these models.

Supplementary Table S4. Selected powers for the pooled multilevel logistic regression models for women aged 25 to 39 years. Each model corresponds to one type of IPV.

IPV type	First power	Second power
Physical	ln(age at first marriage)	ln(age at first marriage) × ln(age at first marriage)
Sexual	(age at first marriage)$^{-1/2}$	ln(age at first marriage)
Psychological	(age at first marriage)$^{-2}$	(age at first marriage)$^{-2}$ × ln(age at first marriage)

Supplementary Table S5. Regression coefficients for the selected powers of the pooled multilevel logistic regression models for women aged 25 to 39 years. Each model corresponds to one type of IPV.

IPV type	Coefficient of the first power (95% CI)	Coefficient of the second power (95% CI)
Physical	5.6 (3.0; 8.2)	-1.1 (-1.5; -0.7)
Sexual	-29.3 (-56.7; -1.9)	-4.0 (-7.2; -0.7)
Psychological	-318.6 (-749.3; 112.1)	148.1 (-49.4; 345.6)
